# Relation between triglycerides and the severity of acute pancreatitis combined with nonalcoholic fatty liver disease: a retrospective study

**DOI:** 10.1186/s12876-023-02951-9

**Published:** 2023-09-14

**Authors:** Lei Zhu, Leyao Yuan, Tingting Wang, Quping Zhu, Qian Zhang, Changbao Pan, Qingcheng Xu, Denghao Deng, Weiwei Chen, Juan Chen

**Affiliations:** Department of Gastroenterology, Northern Jiangsu People’s Hospital, Yangzhou University, No 98, Nantong West Rd, Yangzhou, Jiangsu China 225000

**Keywords:** Acute pancreatitis, Nonalcoholic fatty liver disease, Hypertriglyceridemia, Lipid metabolism

## Abstract

**Background:**

Nonalcoholic fatty liver disease (NAFLD) can exacerbate the severity of acute pancreatitis (AP), and this severity is worsened with increased severity of NAFLD. This study aimed to investigate the relation between serum triglyceride (TG) and the severity of AP with NAFLD by collecting clinical data from AP patients with NAFLD.

**Methods:**

AP patients with NAFLD were divided into 2 groups according to TG levels: hypertriglyceridemia (HTG) group and non-hypertriglyceridemia (NHTG) group.

**Results:**

In total, 598 AP patients with NAFLD were enrolled in this study, including 433 in the HTG group and 165 in the NHTG group. Compared with the NHTG group, AP patients in the HTG group were more serious (*P* < 0.05). The incidence of persistent organ failure (POF), especially persistent respiratory failure, and the ratio of acute peripancreatic fluid collection (APFC) were higher in the HTG group (*P* < 0.05). Higher TG levels were associated with a higher incidence of APFC (*P* < 0.05). Logistic regression analysis showed that the risk of APFC was significantly higher in moderate and severe NAFLD than in mild NAFLD.

**Conclusion:**

HTG may aggravate the severity and local complications of AP combined with NAFLD.

## Introduction

Acute pancreatitis (AP) is a common clinical emergency of the abdomen, and its incidence has been gradually increasing in recent years. The etiology of AP is multifactorial, mainly including biliary, alcoholic, hypertriglyceridemia (HTG), and idiopathic. Biliary disease (45%) and alcoholism (20%) are the most common causes of AP in most high-income countries [1]. Recently, with the improvement of living quality and dietary modifications, the proportion of HTG-AP has gradually increased. There is considerable evidence that the proportion of HTG has surpassed alcoholism in China, becoming the second leading cause of AP [2, 3]. Studies have shown that abnormal lipid metabolism has an important impact on the severity and prognosis of AP [4]. A significant proportion of patients with AP have been clinically found to have nonalcoholic fatty liver disease (NAFLD), a liver disease associated with obesity, insulin resistance, type 2 diabetes (T2DM), hypertension, hyperlipidemia, and metabolic syndrome [5]. NAFLD is a specific manifestation of lipid metabolism abnormalities in the liver, and the incidence is increasing annually [6]. A retrospective study found that NAFLD can exacerbate the severity of AP, which is worsened with the increased severity of NAFLD [4]. As TG is a common risk factor for both, there are few studies on the correlation between the three; thus, this study investigated the relation between TG and the severity of disease in patients with AP combined with NAFLD.

## Methods

### Study population and data collection

This study retrospectively collected the clinical data of 598 patients with AP combined with NAFLD who were hospitalized in Northern Jiangsu People's Hospital from January 1, 2016, to December 31, 2020. It was approved by the ethics committee of the hospital on February 15th, 2023 (No. 2023ky-012) and the protocol complied with the ethical guidelines of the 1975 Declaration of Helsinki. This study has been registered in the Chinese Clinical Trial Registry (Registration number: ChiCTR2300068370). We extracted patients’ information and data from the hospital information system.

### Inclusion and exclusion criteria

The inclusion criteria were as follows: (1) age: 18 years ≤ age ≤ 80 years; (2) met the diagnostic criteria of AP; (3) abdominal pain at admission ≤ 7 days; and (4) NAFLD diagnosed by computed tomography (CT) of the abdomen. The exclusion criteria were: (1) patients with any prior history of any tumor; (2) patients in the advanced or terminal stage of any disease; (3) suffering from alcoholic fatty liver; (4) patients with cirrhosis; (5) pregnant or lactating women; and (6) patients with incomplete patient information.

### Diagnosis of NAFLD

NAFLD was diagnosed by the ratio of computed tomography (CT) values for the liver and spleen, with CT measurements ranging from 88 to 92 mm. Mild NAFLD was defined as a liver/splenic CT ratio of ≤ 1, and the ratio of moderate NAFLD was higher than 0.5 and ≤ 0.7. Severe NAFLD was defined as a ratio ≤ 0.5 [7].

### Hypertriglyceridemia

Hyperlipidemia was defined as serum TG levels ≥ 1.7 mmol/L. The TG level of mild hyperlipidemia was ≥ 1.7 and < 2.3 mmol/L. Moderate hyperlipidemia was defined as a TG level ≥ 2.3 and < 11.2 mmol/L. Severe hyperlipidemia was defined as a TG level ≥ 11.2 and < 22.4 mmol/L. If the TG level was ≥ 22.4 mmol/L, it was defined as very severe hyperlipidemia [8]. Patients’ TG levels were traced to the first detection at the beginning of the onset of AP.

### Local complications

Local complications of AP include acute peripancreatic fluid collection (APFC), acute necrotic collection (ANC), pancreatic pseudocyst (PPC), walled-off necrosis (WON), and infectious pancreatic necrosis (IPN) [9].

### Organ failure

According to the modified Marshall scoring system [10], organ failure was defined by evaluating three organs of the respiratory, cardiovascular and renal systems [9], with a score of no less than two. Transient OF (TOF) was defined as if the OF duration did not exceed 48 h, and persistent OF (POF) was defined as if the OF duration exceeded 48 h.

### Statistical analysis

SPSS 26.0 software was used for statistical analysis. Continuous variable data were expressed using the means ± standard deviation and medians (interquartile range), and categorical variables were expressed as the percentages. Continuous variables were compared using the *t* test or Mann‒Whitney U test. Categorical variable data were tested using the chi-square test or Fisher's exact test. Logistic regression analysis was utilized to screen independent risk factors for APFC in AP patients. *P* < 0.05 was considered statistically significant.

## Results

### Clinical features of the HTG and NHTG groups

**Fig. 1:**
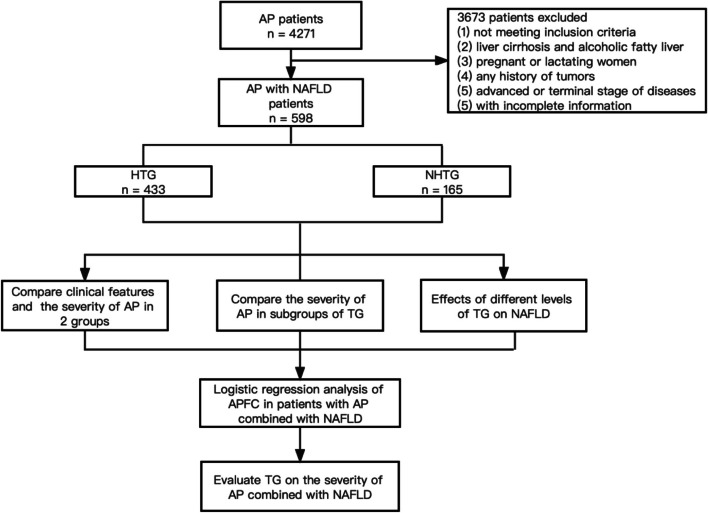
The flow chart of patients’ selection process

In total, 1414 patients diagnosed with AP were included in this study, including 598 patients with NAFLD, 433 patients in the HTG group, and 165 patients in the NHTG group (as shown in the flowchart, Fig. 1). Comparing the demographic and clinical characteristics of the two groups, there were significant differences in terms of age, sex, BMI, WC, and etiology (*P* < 0.05). The mean age of the participants was 46.1 ± 13.9 years, and approximately 64.7% were male. The mean age in the HTG group was younger than that in the NHTG group (42.2 ± 11.5 vs. 55.5 ± 14.8 years) and was dominated by males (70.1%). BMI (26.8 ± 4.0 vs. 25.4 ± 4.1) and WC (90.8 ± 10.9 vs. 86.7 ± 9.8) were significantly higher in the HTG group than in the NHTG group. Regarding the etiology, HTG was the most common cause in the HTG group (67.2%), while gallstones were the most common cause in the NHTG group (64.8%) (Table 1).

**Table 1:** Clinical features of the HTG group and the NHTG group

Variables	Cohort	HTG	NHTG	*p*
Age, (years) (mean + SD)	46.1 ± 13.9	42.2 ± 11.5	55.5 ± 14.8	< 0.001
Sex, (n, %)				< 0.001
Male, (n, %)	387 (64.7)	303 (70.1)	84 (51.4)	
Female, (n, %)	211 (35.3)	130 (29.9)	81 (48.6)	
BMI(kg/m2)	26.4 ± 4.1	26.8 ± 4.0	25.4 ± 4.1	< 0.001
WC(cm)	89.6 ± 10.8	90.8 ± 10.9	86.7 ± 9.8	< 0.001
Etiologies, (n, %)				< 0.001
gallstones	170 (28.4)	63 (14.5)	107 (64.8)	< 0.001
HTG	291 (48.7)	291 (67.2)	0 (0)	< 0.001
Alcoholic	8 (1.3)	7 (1.6)	1 (0.6)	0.455
ERCP	0 (0)	0 (0)	0 (0)	1.00
Idiopathic	114 (19.1)	63 (14.5)	51 (30.9)	< 0.001
Mixedness	11 (1.8)	6 (1.4)	5 (3.0)	0.47
Others	4 (0.7)	3 (0.7)	1 (0.6)	1.00

### Comparison of the severity of AP in the HTG and NHTG groups

According to the 2012 revision of Atlanta classification of AP [9], there were 273 (45.7%), 294 (49.2%) and 31 (5.2%) patients with mild AP (MAP), moderately severe AP (MSAP), and severe AP (SAP), respectively, in this study. Compared with the NHTG group, the incidence of MAP was lower in the HTG group (52.1% vs. 43.2%). Comparisons between the two groups revealed that the incidence of MSAP (50.3% vs. 46.1%) and SAP (6.5% vs. 1.8%) was significantly higher in the HTG group than in the NHTG group (*P* < 0.05). In terms of OF, the incidence of POF (6.5% vs. 1.8%), especially persistent respiratory failure (5.3% vs. 1.2%), was higher in the HTG group than in the NHTG group (*P* < 0.05). However, there were no significant differences in the incidence of persistent heart failure, persistent renal failure, or TOF between the two groups (*P* > 0.05). In terms of local complications, the HTG group was more prone to APFC than the NHTG group (52.2% vs. 42.2%, *P* < 0.05). In addition, in regard to biochemical indices, the TG and WBC levels in the HTG group were significantly higher than those in the NHTG group (*P* < 0.05), and the HDL-C levels were significantly lower than those in the NHTG group, indicating that the HTG group was more severely affected (Table 2).

**Table 2:** Comparison of the severity of AP in the HTG group and NHTG group

Variables	Cohort	HTG	NHTG	*P*
Atlanta classification (n, %)				0.019
MAP	273 (45.7)	187 (43.2)	86 (52.1)	
MSAP	294 (49.2)	218 (50.3)	76 (46.1)	
SAP	31 (5.2)	28 (6.5)	3 (1.8)	
OF, (n, %)				
POF	31 (5.2)	28 (6.5)	3 (1.8)	0.022
Persistent respiratory failure	25 (4.2)	23 (5.3)	2 (1.2)	0.022
Persistent renal failure	14 (2.3)	13 (3.0)	1 (0.6)	0.127
Persistent heart failure	1 (0.2)	1 (0.2)	0 (0)	1.000
TOF	18 (3.0)	13 (3.0)	5 (3.0)	0.968
Local Complications (n, %)				
APFC (n, %)	294 (49.2)	226 (52.2)	68 (41.20)	0.016
ANC (n, %)	26 (4.3)	18 (4.2)	8 (4.8)	0.711
PPC (n, %)	13 (2.2)	10 (2.3)	3 (1.8)	0.713
WON (n, %)	3 (0.5)	3 (0.7)	0 (0)	0.565
IPN (n, %)	2 (0.3)	2 (0.5)	0 (0)	0.382
MCTSI	4 (2–6)	4 (2–6)	4 (2–6)	0.296
Laboratory indicators				
TG	4.5 (1.7–15.5)	8.7 (3.5–21.7)	1.0 (0.7–1.4)	< 0.001
LDH	270.5 (208.8–431.1)	270.0 (205.8–429.3)	271.0 (211.5–436.5)	0.717
HDL-C	0.9 (0.7–1.2)	0.8 (0.6–1.1)	1.2 (0.9–1.5)	0.000
WBC	12.2 (9.4–15.1)	12.5 (10.0–15.5)	10.7 (7.9–13.9)	0.003
BUN	4.9 (3.7–6.3)	4.9 (3.8–6.3)	5.1 (3.5–6.5)	0.052
CRE	72.0 (60.0–84.0)	72.0 (60.0–84.8)	72.0 (59.8–82.0)	0.110
Ca^2+^	2.2 (2.1–2.3)	2.2 (2.1–2.3)	2.2 (2.1–2.3)	0.891

### Comparison of the severity of AP in the subgroups of TG

According to the TG levels, the HTG group was further subdivided into four subgroups: (1) 1.7 ≤ TG < 2.3 44 cases; (2) 2.3 ≤ TG < 5.65 125 cases; (3) 5.6 ≤ TG < 22.4 163 cases; (4) TG ≥ 22.4 101 cases. There was no statistically significant difference in the incidence of OF among the four groups (*P* > 0.05). With increasing TG levels, the incidence of APFC gradually increased (45.5% vs. 48.8% vs. 50.9% vs. 61.4%, *P* < 0.001), while the incidence of other local complications was not different (*P* > 0.05) (Table 3).

**Table 3:** Comparison of the severity of AP in the subgroups of TG

Variables	1.7–2.3	2.3–5.65	5.65–22.4	≥ 22.4	*p*
Local Complications (n, %)					
APFC (n, %)	20 (45.5)	61 (48.8)	83 (50.9)	62 (61.4)	0.043
ANC (n, %)	2 (4.5)	2 (1.6)	7 (6.1)	7 (6.9)	0.114
PPC (n, %)	1 (2.3)	3 (2.4)	3 (1.8)	3 (3.0)	0.849
WON (n, %)	0 (0)	0 (0)	1 (0.6)	2 (2.0)	0.109
IPN (n, %)	1 (2.3)	0 (0)	1 (0.6)	0 (0)	0.458
OF (n, %)					
POF (n, %)	6 (13.6)	6 (4.8)	12 (7.4)	4 (4.0)	0.218
TOF (n, %)	1 (2.3)	5 (4.00)	4 (2.5)	3 (3.0)	0.804

### Effects of different levels of TG on NAFLD

According to the ratio of CT values for the liver and spleen, the patients were further divided into mild-NAFLD (429 cases) and moderate-severe NAFLD (M + S-NAFLD) (169 cases). The effects of different levels of TG on the severity of NAFLD were analyzed, and it was found that, when TG ≥ 5.65, the proportion of moderate-severe NAFLD was higher (57.4% vs. 38.9%, *P* < 0.001), demonstrating that NAFLD is relatively more severe when TG is higher (Table 4).

**Table 4:** Effects of different levels of TG on NAFLD

TG	Cohort	Mild-NAFLD	M + S-NAFLD	*P*
1.7–2.3 (n, %)	44 (7.4)	34 (7.9)	10 (2.3)	0.449
2.3–5.65 (n, %)	125 (20.9)	87 (20.3)	38 (22.5)	0.550
≥ 5.65 (n, %)	264 (44.1)	167 (38.9)	97 (57.4)	< 0.001

### Logistic regression analysis of APFC in patients with AP combined with NAFLD

Sex, age > 60 years, BMI, TG, hypertension, diabetes, coronary heart disease, recurrence ≥ 2 times, and severity of NAFLD were included in the logistic regression model, with APFC as the dependent variable. The univariate logistic regression model showed that BMI, TG, diabetes, moderate NAFLD, severe NAFLD, and AP recurrence ≥ 2 times were risk factors for APFC in patients with AP combined with NAFLD. Further incorporating the factors with *P* < 0.2 into the multivariate logistic regression model, the results showed that BMI (OR = 0.947, 95% CI 0.906–0.990), TG (OR = 1.017, 95% CI 1.007–1.028), recurrence ≥ 2 times (OR = 1.702, 95% CI 1.030–2.813), moderate NAFLD (OR = 2.353, 95% CI 1.280–4.323), and severe NAFLD (OR = 3.252, 95% CI 1.623–6.516) were independent risk factors, among which the risk of APFC in patients with moderate NAFLD was 2.353 times that of mild patients, and the risk of APFC in patients with severe NAFLD was 3.252 times that of mild patients (Table 5).

**Table 5:** Logistic regression analysis of APFC in patients with AP combined with NAFLD

Variable	Univariate		multivariate	
	OR (95%CI)	P	OR(95%CI)	P
Age > 60	0.960 (0.631–1.461)	0.849		
Male	0.964 (0.689–1.348)	0.829		
BMI	0.950 (0.912–0.990)	0.015	0.947 (0.906–0.990)	0.016
TG	1.016 (1.006–1.027)	0.001	1.017 (1.007–1.028)	0.001
Recurrence ≥ 2 times	1.434 (0.894–2.299)	0.135	1.702 (1.030–2.813)	0.038
Hypertension	0.881 (0.623–1.245)	0.473		
Diabetes	0.699 (0.467–1.047)	0.083	0.816 (0.527–1.264)	0.363
Coronary heart disease	0.723 (0.160–3.257)	0.672		
Mild-NAFLD				
Moderate-NAFLD	2.190 (1.236–3.882)	0.007	2.353 (1.280–4.323)	0.006
Severe-NAFLD	3.368 (1.734–6.543)	< 0.001	3.252 (1.623–6.516)	0.001

## Discussion

AP is a common clinical gastrointestinal emergency with a progressively increasing incidence worldwide [11]. Therefore, a rapid and accurate assessment of the severity of AP is important for appropriate treatment and supportive management [12]. As all we know, HTG is one of the most important etiologic in AP. Elevated TG levels are associated with severity and prognosis of AP, including pancreatic necrosis, organ failure, and so on. As the increasing of TG severity grades, the incidence of localized complications in patients increases significantly and AP becomes more severe [13–15]. With the improvement of living standards and the adjustment of dietary structure, NAFLD has become the most common liver disease in clinical practice [16]. It has been shown that NAFLD is a risk factor for AP [17], which can aggravate the severity of AP disease.

However, the risk factors for NAFLD aggravating AP severity have not yet been definitely identified by previous studies; therefore, this study aimed to explore the relation between TG and the severity of AP combined with NAFLD. Our study demonstrated that HTG aggravates the severity of AP patients with NAFLD, and TG was an independent risk factor for local complications. Moreover, higher serum TG levels were associated with a higher risk of local complications, suggesting that TG may play an important role in the severity of AP with NAFLD.

Young men were predominant in the HTG group, which is consistent with the clinical characteristics of NAFLD and HTG-AP [16, 18]. The WC and BMI of the HTG group were significantly higher than those of the NHTG group, which may be related to the poor lifestyle habits of young males, and such populations are often accompanied by a high-oil and high-fat diet, staying up late, lack of exercise, and alcohol abuse. Thus, they are more likely to have abnormal lipid metabolism, eventually leading to abdominal obesity. Previous studies have found that obesity is an important risk factor for NAFLD [19], and abdominal obesity is also an independent risk factor for AP [20].

In terms of primary outcomes, the results of this study revealed that the severity of AP was higher in the HTG group than in the NHTG group, as shown by a significantly higher proportion of moderately severe and severe AP in the HTG group than in the NHTG group, as well as a higher incidence of OF and local complications, especially POF and APFC. Meanwhile, in the TG subgroup analysis, the incidence of APFC gradually increased with higher TG levels, indicating that higher TG levels in AP patients with NAFLD may be associated with more severe disease and a relatively worse prognosis. A global epidemiological study on dyslipidemia by Pirillo et al. suggested that HTG is associated with the severity of NAFLD and AP [21]. The study also found that the incidence of moderate and severe NAFLD was also higher when TG was elevated. A clinical study by Mikolasevic et al. found that the presence of NAFLD indicates a higher risk of developing more severe AP and may serve as an additional prognostic tool [12]. TG has an exacerbating effect on both NAFLD and AP severity, and logistic regression analysis also showed that TG is an independent risk factor for APFC in AP patients with NAFLD.

In this study, AP recurrence ≥ 2 times was also identified as a risk factor for APFC. HTG and hypercalcemia are metabolic causes of AP recurrence [22]. Familial chylomicronemia syndrome (FCS) has previously been shown to be associated with an increased risk of AP recurrence, and NAFLD was commonly observed in patients with FCS [23]. The manifestation of FCS is a large accumulation of serum TG, and it can therefore be speculated that elevated serum TG levels increase the risk of developing NAFLD, further increasing the risk of AP recurrence and thus aggravating the condition of AP.

The mechanism by which TG in NAFLD exacerbates the severity of AP has not yet been clarified and needs to be further explored. At present, the possible mechanisms mainly include the following. First, obesity leads to organ steatosis and altered serum adipokines, and an abnormal adipokine environment leads to increased tissue infiltration of monocytes and macrophages, thereby producing proinflammatory cytokines that alter organ function [24]. In addition, as an endocrine organ, adipose tissue can release a variety of adipokines such as adiponectin, resistin, and leptin, which are involved in the occurrence and development of AP. Studies have found that resistin can increase TG levels, which can increase free fatty acid (FFA) levels and also induce hepatic steatosis, thus aggravating abnormal lipid metabolism. The reduced level of adiponectin in the blood of obese patients can reduce FFA metabolism, while its ability to inhibit TNFα decreases, thereby enhancing the body's inflammatory response and aggravating local damage to the pancreas [25, 26]. Last, fatty liver is often associated with HTG, which leads to free radical accumulation, microcirculation disturbances, oxidative stress, and acinar necrosis in AP [27].

This study has several limitations nevertheless. Firstly, as a single-center retrospective study, further validation by multicenter studies is needed. Additionally, as liver biopsy is still the gold standard for describing liver histological changes in patients with NAFLD [28], there are certain errors in the diagnosis of NAFLD by using CT values for the liver and spleen. Furthermore, most of the TG data were collected at admission, which may not reflect the patient's real TG level at the onset of AP, possibly introducing bias.

## Conclusion

In conclusion, this study showed that TG is closely related to the severity and local complications in patients with AP complicated with NAFLD and is an independent risk factor for the development of local complications.

## Data Availability

All data used to support the findings of this study are available from the corresponding author upon request.

## Abbreviations

AP: Acute pancreatitis
NAFLD: Nonalcoholic fatty liver disease
HTG: Hypertriglyceridemia
NHTG: Non-hypertriglyceridemia
CT: Computed tomography
BMI: Body Mass Index
WC: Waist circumference
ERCP: Endoscopic retrograde cholangiopancreatography
OF: Organ failure
POF: Persistent organ failure
TOF: Transient organ failure
APFC: Peripancreatic fluid collection
ANC: Acute necrotic collection
PPC: Pancreatic pseudocyst
WON: Walled-off necrosis
IPN: Infectious pancreatic necrosis
HTG-AP: Hypertriglyceridemia acute pancreatitis
MAP: Mild acute pancreatitis
MSAP: Moderately severe acute pancreatitis
SAP: Severe acute pancreatitis
M + S-NAFLD: Moderate-severe NAFLD
FFA: Free fatty acid
FCS: Familial chylomicronemia syndrome
